# Exome-based cancer driver gene comprehensive testing can provide a genetic diagnosis for individuals with triple-negative breast cancer

**DOI:** 10.1371/journal.pone.0356762

**Published:** 2026-08-24

**Authors:** Daniel Alzate, Angel Yobany Sánchez, Yovana Pacheco, Mario Isaza Ruget, Ramiro Sánchez, Carolina Castillo, Carlos A. Parra-López

**Affiliations:** 1 Immunology and Translational Medicine Group, Faculty of Medicine, Universidad Nacional de Colombia, Bogotá, Colombia; 2 PhD Programme in Oncology, Faculty of Medicine, Universidad Nacional de Colombia, Bogotá, Colombia; 3 Department of Pathology, Faculty of Medicine, Universidad Nacional de Colombia, Bogotá, Colombia; 4 Clinical Pathology Research Group (INPAC), Research Unit, Fundación Universitaria Sanitas, Bogotá, Colombia; 5 Clínica del Seno, Bogotá, Colombia; 6 Microbiology Department, Faculty of Medicine, Universidad Nacional de Colombia, Bogotá, Colombia; Odense University Hospital, DENMARK

## Abstract

Triple-negative breast cancer (TNBC) is characterized by aggressive behaviour, high tumor heterogeneity, and an increased likelihood of recurrence and early metastasis. These factors hinder successful treatment. Genetic diagnosis enables personalized clinical recommendations and treatment options. The objective of this study was to validate whole-exome sequencing (WES) and variant prioritization in cancer susceptibility genes (CSG) associated with hereditary cancer (HC) predisposition in TNBC patients (n = 24). We present the development of a reproducible bioinformatic pipeline and its technical validation in a validation cohort (n = 25). This cohort comprised individuals with diverse primary tumors who had a previously confirmed molecular diagnosis of a hereditary cancer syndrome, serving as gold-standard cases to assess the pipeline’s analytical accuracy. We consolidated a comprehensive panel of cancer genes and determined all variants in the TNBC discovery cohort (12.5% of patients), identifying three pathogenic germline variants (gPV) in *ATM, RAD51D, and BRCA1*. These genes are involved in the molecular pathway of DNA repair by homologous recombination (HRD). Our results demonstrate that the developed bioinformatic pipeline provides reliable genetic diagnosis of cancer predisposition syndromes from exome data, applicable not only to TNBC patients but also to individuals with any cancer suspected of having a hereditary component.

## Introduction

Triple-negative breast cancer (TNBC) accounts for approximately 15% of all breast cancers and is generally very aggressive, with more frequent distant spread, high tumor heterogeneity, higher recurrence, and earlier metastasis, all of which hinder the success of new therapies [1]. TNBC often occurs in women under 40 years of age and has a mortality rate of 40% within the first 5 years after diagnosis, a median survival after metastasis of only 13.3 months, and a recurrence rate after surgery of 25% [2]. The predominant systemic therapy for most metastatic TNBC (mTNBC) is chemotherapy, but responses are often short-lived, and patients have a median overall survival of 12–18 months [3].

Hereditary cancer (HC) accounts for 5% to 10% of all cancers, although it may be underdiagnosed in less genetically studied populations [4]. Criteria for suspecting HC include the development of multiple primary tumors, clustering of affected family members with cancer, and/or early age at tumor onset [5]. Identifying variants in well-established cancer susceptibility genes (CSG) and in candidate genes that increase cancer risk is critical for understanding the pathophysiology and advancing the development of new precision therapies and early interventions. Therefore, it is urgent to conduct diagnostic testing actively to identify patients and family members who may carry a harmful variant associated with a hereditary cancer syndrome. This will help establish a personalized follow-up plan to detect cancer early, increasing the likelihood of successful treatment.

Tests using smaller genetic panels have the drawback that the causal variant may be missed, especially when there is no clear phenotypic sign, which often occurs in individuals with different primary tumors. Conducting diagnoses based on the whole exome enables the identification of variants in well-established CSG, as well as in emerging candidate genes. This approach would enable the identification of pathogenic or likely pathogenic (**P/LP)** causal variants and elucidate genotype-phenotype associations for other rare and less-studied genetic diseases [6]. In this study, we investigated germline genetic variants from the exome, focusing on a comprehensive and expanded panel of cancer genes that we consolidated (n = 813 genes). We validated a reproducible pipeline with a cohort of patients (n = 25) who had clinical and molecular diagnoses of HC (pipeline validation cohort), and we applied it to patients with TNBC (TNBC discovery cohort, n = 24). This tumor type was selected for its molecular heterogeneity, aggressive phenotype, and limited sensitivity of standard gene panels in detecting rare susceptibility variants, and as a proof of concept for the usefulness of whole-exome sequencing (WES) to overcome this limitation, particularly in underrepresented populations such as those of Latin American ancestry.

## Materials and methods

### Ethical approval and consent to participate

All participants gave their informed consent, and the study was approved by the local ethics committee of the National University of Colombia (study number 2019–5738). Although in the present study the secondary findings report was not performed due to the approved protocol restrictions by the ethics committees, the technical and bioinformatics pipeline feasibility was demonstrated. This study was conducted in accordance with the ethical standards described in the Declaration of Helsinki.

### Study cohorts

The first study cohort consists of 25 individuals with hereditary cancer syndromes who met clinical criteria and had a molecular diagnosis, i.e., a germline P/LP variant that explains the patients’ phenotype. A total of 25 cases meet these criteria and are hereafter referred to as the “pipeline validation cohort.” Our second group, called the “TNBC discovery cohort,” includes 24 patients with a confirmed pathological diagnosis of TNBC. Both groups underwent whole exome sequencing (WES) after signature and acceptance of written informed consent for research.

### Whole exome sequencing of germline DNA from blood

Genomic DNA was extracted from peripheral blood, and the DNA sample was quantified by spectrophotometry (Nanodrop OneC) and fluorometry (Qubit 4) to normalize the DNA concentration across samples. Whole Exome Sequencing (WES) libraries were prepared using enrichment kits according to the manufacturer’s instructions (*Agilent SureSelect All Exon V6 for the TNBC discovery cohort and Illumina DNA Prep with Exome 2.5 Enrichment for the pipeline validation cohort*). Briefly, DNA was randomly fragmented into 180–280 bp fragments. The resulting fragments were end-repaired and ligated with Illumina molecular adapters. Adapter-containing fragments were amplified by PCR, size-selected, and purified. Hybridization capture of the libraries was performed using a buffer containing biotin-labelled probes, and streptavidin-coated magnetic beads were used to capture the gene exons. Subsequently, unhybridized fragments were washed away, and the probes were digested. The captured libraries were further enriched by PCR amplification. Libraries were analysed with Qubit and a fragment bioanalyzer to determine size distribution. They were then quantified, pooled, and sequenced on Illumina platforms using a 2x150 bp paired-end read strategy.

### Development of a pipeline for germline variant detection based on *GATK* best practices

The study evaluates the feasibility and proof of concept for implementing an end-to-end bioinformatics pipeline. This workflow integrates the recommendations of GATK Best Practices [7] to establish a robust and reproducible pipeline for germline variant identification, covering everything from initial data quality assessment to variant annotation and prioritization. First, the quality assessment was performed with FastQC [8], which detects potential contamination or artifacts before proceeding with alignment. For sequence alignment, the cleaned data are mapped to the reference genome (hg38) using the Burrows-Wheeler Aligner *BWA-MEM* [9] software, and the output files are converted and sorted by chromosomal position (SAM/BAM) with Samtools (view, sort, index). GATK then performs critical steps for variant calling optimization, including duplicate marking (*MarkDuplicatesSpark)*, alignment sorting (*SortSam)*, base quality recalibration (*BaseRecalibrator, ApplyBQSR)*, and finally, germline variant calling with *HaplotypeCaller* [10]. After separating SNPs and INDELs with *SelectVariants,* quality filters are applied using the *VariantFiltration* tool to obtain a set of reliable variants. The SNP and InDel filter parameters are shown below: SNP: *QD* < 2.0, *FS* > 60.0, *MQ* < 40.0, *HaplotypeScore*>13.0, *MappingQualityRankSum* < −12.5, *ReadPosRankSum* < −8.0; InDel: *QD* < 2.0, *FS* > 200.0, *ReadPosRankSum* < −20.0. For general metrics, variants with a depth > 20x and an allele frequency>15% were considered valid. In a complementary manner, alignment metrics (*CollectAlignmentSummaryMetrics)* and library fragment size metrics (*CollectInsertSizeMetrics)* are generated with GATK to evaluate the consistency of the process. The resulting variants are functionally annotated with *Ensembl VEP* [11] and the *Franklin Genoox* platform, incorporating information on the allele frequency of the variants, molecular effects, and clinical databases. *Databases used in variant annotation include: dbSNP, gnomAD (Exome), gnomAD (Genome), Sift, Polyphen2, Mut Taster, Mut Assessor, Fathmm, ClinVar, Gerp, Revel, SpliceAI, Vep, dbNSFP, dbscSNV. **Genomic variant classification criteria: The guidelines of the American College of Medical Genetics and Genomics (ACMG) [12] and the Association for Clinical Genomic Science (ACGS) [13] were followed.

To ensure reproducibility, this workflow was implemented in a Docker container with specific versions of the aforementioned bioinformatics tools. Finally, we tested the bioinformatics pipeline using a validation set of independent samples previously sequenced and analysed, with platforms developed for a clinical diagnostic environment (*Euformatics Genomics Hub*: https://www.euformatics.com, *Emedgene*:https://emg.emedgene.com, *Franklin:* https://franklin.genoox.com), based on fastq files from patients for whom a germline variant previously identified, responsible for a hereditary cancer syndrome, had been previously identified. Then, we ran the raw data (fastq) from all these patients through the new germline pipeline, and 100% concordance was obtained in the results, confirming the variants previously identified with the pipeline and the clinical exome analysis platform. The concordance strictly refers to the ability of the bioinformatics pipeline to accurately detect and annotate the causal variants of the “gold standard” phenotype present in the pipeline validation cohort. These results confirm the robustness and reliability of this pipeline for germline variant identification in the context of precision genomic diagnosis.

Code availability and hybrid Docker orchestration (high reproducibility): The exact versions of the bioinformatics tools, the complete source code, the command-line parameters, and the computational environments are available in the GitHub repository at https://github.com/GatoconBata-07/Germinal_Pipeline_GATK_BEST.

### Consolidation of an expanded and comprehensive panel of cancer susceptibility genes

To prioritize germline variants with potential impact on cancer predisposition, genes were selected from a panel of cancer susceptibility genes (CSG) compiled from the literature [14–17], and from gene panels used in clinical diagnosis (Illumina TruSight Oncology, Pan-cancer panel CD Genomics). We selected the genes from the IntOGen database that were present in at least 1% of cancer patients. We also selected genes that were consensus in at least three sources in the *OnkoKB* database, and were cited as cancer drivers. We finally obtained a comprehensive and expanded panel of 813 genes (*pancancer panel*).

### Prioritization of germline variants

After exome annotation, germline variants with a sequencing depth of ≥20 reads and an allelic frequency >15% were selected from the comprehensive panel.The following criteria were used for variant prioritization: i) variants predicted to cause loss of function (nonsense, frameshift, and canonical splice sites: + /-3 bp); ii) missense variants and in-frame indels; iii) synonymous variants. The terms were entered into the Franklin platform using the Human Phenotype Ontology (HPO) nomenclature: Tumor (HP:0002664), Carcinoma (HP:0030731), Breast cancer (HP:0003002). Finally, the prioritized variants were interpreted according to the American College of Medical Genetics and Genomics (ACMG) criteria [12].

## Results

### The developed pipeline enables precise identification of causal variants in patients with various types of cancer

**Fig 1:**
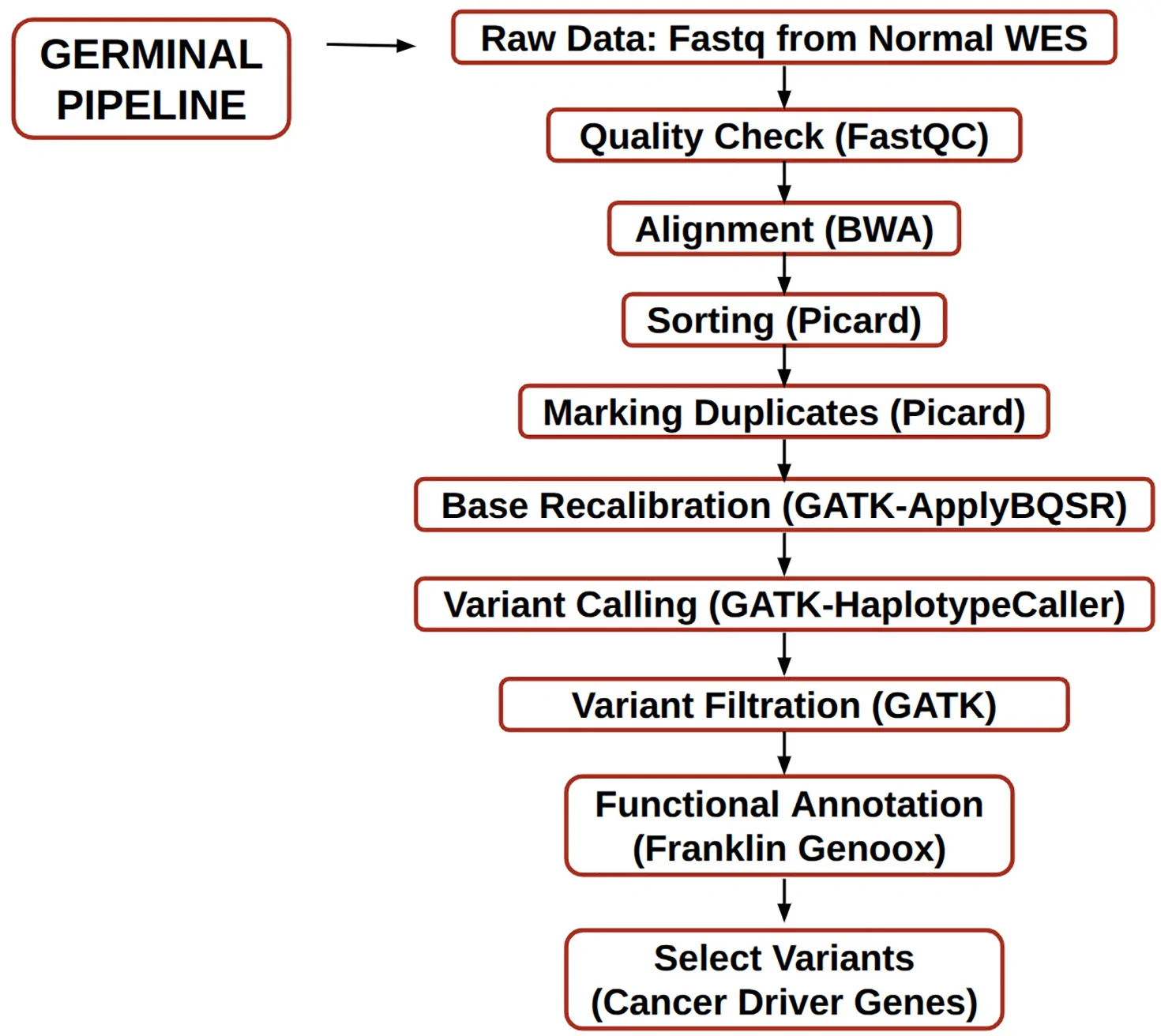
Bioinformatics pipeline for the identification of germline variants in cancer susceptibility genes.

Using complete exome data from cancer patients, we developed a pipeline (Fig 1) to identify germline variants in accordance with the Broad Institute´s best practices (*GATK*), and we confirmed the results using a pipeline validation cohort comprising samples previously characterized by a pipeline used in clinical diagnostic practice. This validation set identified the causal P/LP variants for various hereditary cancers in 25 patients, including breast cancer, tuberous sclerosis, juvenile myelomonocytic leukemia, colorectal cancer, ovarian cancer, multiple osteochondromas, schwannomatosis, neurofibromatosis, familial adenomatous polyposis, and Li-Fraumeni syndrome. It revealed a spectrum of P/LP variants in the *BRCA1, BRCA2, TSC2, MLH1, TP53, PALB2, KRAS, STK11, PMS2, EXT1, BAP1, NF1,* and *NF2* genes.

Based on validation cohort results (n = 25 patients), summarized in S3 Table, we identified 25 causal variants associated with cancer in patients, of which 23 (92%) are classified as pathogenic and 2 (8%) are likely pathogenic. Truncating events that result in loss of function, dominated—frameshift 11/25 (44%) and nonsense 7/25 (28%)—followed by missense 5/25 (20%) and canonical splice site 2/25 (8%). Most pathogenic variants were found in genes involved in the homologous recombination DNA repair pathway (HDR): (*BRCA1/BRCA2/PALB2* (13/25: 52%), followed by genes involved in the mismatch repair pathway (*MLH1/PMS2*, 3/25: 12%). and pathogenic variants were less frequently identified in other cancer predisposition syndromes, such as Li-Fraumeni syndrome, Peutz-Jeghers syndrome, tuberous sclerosis, and neurofibromatosis (*TP53, STK11, TSC2, NF1/NF2, BAP1, EXT1, KRAS*) among others. This pattern—characterized by the inactivation of tumor suppressors and the prevalence of truncating variants in *BRCA1/BRCA2/PALB2*—is consistent with population studies indicating that protein-truncating variants in these genes significantly increase cancer risk and form the core of susceptibility to hereditary breast and ovarian cancer [18].

Overall, the results of the pipeline developed for germline variant identification show an exact match in detecting P/LP variants previously identified by a clinical diagnostic bioinformatics platform. These findings have significant clinical implications, including genetic counselling and monitoring family members for early follow-up and intervention for carriers of the identified P/LP variants.

### The whole-exome approach enables the detection of germline variants in cancer-susceptibility genes in TNBC patients

**Fig 2:**
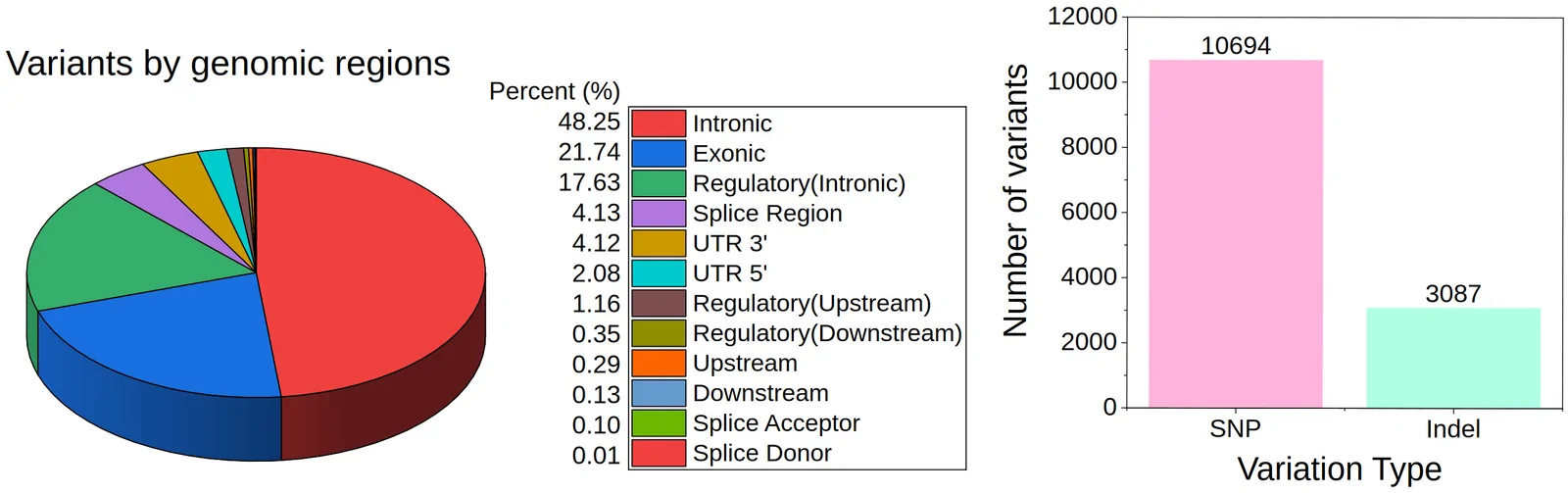
Genomic distribution of variants found in the *pancancer* panel. (A) Among all variants, 48.25% are in intronic regions, 21.74% in exons, and 17.63% in intronic regulatory regions. Variants in splicing regions make up 4.13% and variants in the 3′ and 5′ UTRs account for 4.12% and 2.08%, respectively, potentially affecting RNA maturation and translation. An additional 1.16% and 0.35% are located in upstream and downstream regulatory regions; other variants are mapped to these regions (0.29% and 0.13%). Canonical splice sites contain the fewest variants (acceptor, 0.10%; donor, 0.01%). Overall, most germline variants are found in non-coding regions. The high number of variants annotated as benign or likely benign (11,072/13,781) within *pancancer* genes indicates they are polymorphisms without direct roles in tumor development. (B) Counts and types of alterations in *pancancer* genes. SNV, single-nucleotide variant; indel, insertion/deletion.

**Fig 3:**
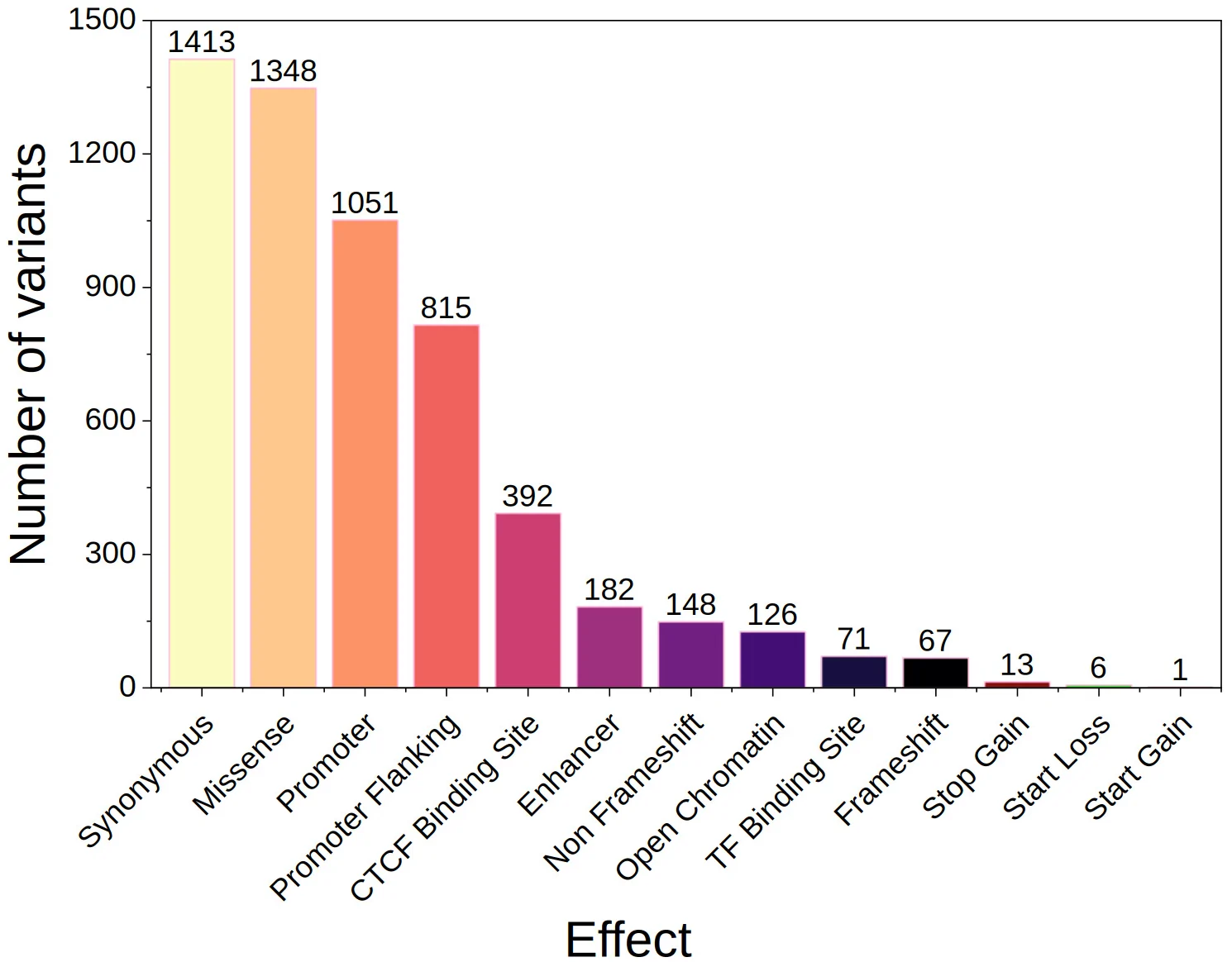
Analysis of variant distribution based on their functional effects in cancer genes identified in TNBC patients. The analysis shows that most variants are synonymous (1,413), followed by missense (1,348), and promoter region variants (1,051). Variants in promoter-flanking regions (815) and CTCF-binding sites (392) may influence epigenetic and transcriptional regulatory mechanisms. Additionally, variants were found in enhancers (182), open chromatin regions (126), and TF binding sites (71), suggesting a potential effect on chromatin accessibility. Non-frameshift variants (148), frameshift variants (67), and stop-gain mutations (13) can alter the length or integrity of the protein reading frame. In contrast, start-loss (6) or start-gain variants involve the loss or gain of the translation start codon. The remaining variants are mostly located in deep intronic regions, making their functional effects unclear. These findings emphasize the importance of studying both coding variants and those in genome regulatory regions.

After achieving 100% concordance in the pipeline validation cohort, we sequenced the whole exome of TNBC patients (TNBC discovery cohort) and processed the data through the pipeline. We analyzed all germline variants in a panel of 813 cancer genes (*pancancer panel*) (Fig 2 and S4 Table). A total of 13,781 variants were identified in these patients within the panel, including 10,694 single-nucleotide variants (SNVs) and 3,087 insertions/deletions (InDels) (S5 Table). Among these variants, 11,072 are benign or probably benign, 2,702 are variants of uncertain clinical significance, and 7 are P/LP variants, with only 3 explaining the hereditary cancer phenotype in patients with TNBC. We reviewed all variants in the *pancancer panel and* established the functional consequences for all patients (Fig 3).

We identified three pathogenic variants associated with an increased breast cancer risk in three patients: in high-risk genes *BRCA1* p.Gly559Valfs*13 (Patient 21: P21), *RAD51D* p.Val32Phefs*38 (Patient 9: P9), and a moderate-risk gene in *ATM* p.Lys750= (Patient 6: P6). These variants affect genes involved in the homologous recombination repair (HRR) pathway. Recent studies show that *BRCA1* and *RAD51D* are high-risk genes that predispose individuals to breast cancer. *ATM* mutations are linked to an increased risk of breast, pancreatic, and lymphoma cancers due to loss of function, which can lead to the accumulation of mutations [19].

The variant in the *ATM* gene (ATM:c.2250G > A, p.Lys750 = NM_000051.4) was particularly interesting to us because, despite its moderate risk classification, it has been observed in patients with hereditary cancer. A case reported in the literature [20] describes a 49-year-old female diagnosed with breast cancer who carries this variant. Expert panels such as ClinGen and Decipher have identified associations of P/LP variants in the *ATM* gene with hereditary breast carcinoma and familial ovarian cancer, as supported by publicly available resources [21,22]. Additionally, this variant has 28 entries in the ClinVar database [23], all classified as P/LP (Accession: VCV000003044.76). The variant affects a critical position at the splice donor site in exon 14 (S7 Information). *In silico* splice prediction tools indicate a strong disruptive or deleterious effect (SpliceAI = 0.94). Consistent with this, functional studies [24] demonstrate that ATM:c.2250G > A alters normal splicing and results in complete skipping of exon 14, producing the r.2125_2250del transcript and a 42-amino acid frameshift deletion (p.Glu709_Lys750del).

Classic studies in lymphoblasts show that variants affecting this canonical splicing site reduce ATM protein expression, decrease kinase activity, and increase chromosomal instability, supporting a deleterious effect at the molecular level [25]. Overall, the evidence combines robust *in silico* predictions, RNA validation, and functional data pointing to a pathogenic mechanism of splicing alteration caused by a synonymous variant at the end of exon 14. This is a pathogenic mechanism inferred from the literature, not a functional validation derived directly from the *ATM* variant patient.

After reviewing the ACMG criteria for variants classified as P/LP and recategorizing them, we identified variants that do not causally explain the phenotype of TNBC patients (S6 Table). These variants include *SDHA* c.1064 + 2T > A (LP), *RUNX1* p.Arg427Profs*174 (LP), *FANCA* p.Arg1187Glufs*28 (P), and *FBXO11* p.Gln21Serfs*116 (LP). Deleterious variants in the *SDHA* gene are linked to pheochromocytomas, paragangliomas, and gastrointestinal stromal tumors [26]. P/LP variants in the *RUNX1* gene are linked to hematologic malignancies [27], such as myelodysplastic syndromes, while biallelic variants in the *FANCA* gene are associated with Fanconi anemia and a significant predisposition to leukemia and squamous cell carcinomas [28]. Alterations in the *FBXO11* gene have been linked to diffuse large B-cell lymphomas [29]. These findings are not part of the set of genes with causal evidence for breast cancer risk and are therefore considered unrelated to the development of TNBC at this time.

## Discussion

The contribution of this work is the WES integration of an end-to-end bioinformatics pipeline based on the GATK best-practices workflow to identify variants in cancer susceptibility genes (CSG) and new variants that may confer risk for TNBC and other tumors. We anticipate that, beyond identifying variants in CSG, WES enables comprehensive detection of rare and atypical variants that targeted approaches miss. The main value consists in the design, development, and validation of a fully independent, open-source bioinformatics methodology. Unlike studies that rely on restrictive commercial licenses and limited genetic panels, our study provides a whole-exome framework focused on 813 cancer-driving genes. By integrating BWA-MEM, GATK best practices, and Ensembl VEP into a Docker container, we offer a highly reproducible infrastructure that ensures analytical transparency and sovereignty over the data and its interpretation. We highlight that this open-source platform offers 100% compatibility with clinical-grade diagnostic platforms and integrates a flexible workflow suitable for breast cancer patients with suspected hereditary components, providing a clear and auditable path from raw exome data to clinically relevant diagnostic results.

To evaluate the utility of WES versus standard tests, we compared the diagnostic performance of three modalities in our TNBC cohort. The hypothetical application of a panel restricted to *BRCA1/2* would have identified a causative variant in 4.1% of patients (1/24). The use of an extended panel for hereditary breast cancer (based on curated lists such as *PanelApp* or standard commercial panels) would have increased the diagnostic performance to 12.5% (3/24), a value consistent with expected rates for hereditary TNBC. In contrast, our WES approach successfully identified variants in these three patients and, additionally, detected pathogenic or probably pathogenic (P/LP) germline variants in three other patients (involving the *SDHA, RUNX1*, and *FBXO11* genes; S6 Table). While targeted panels may offer lower initial costs and slightly faster turnaround times, WES has long-term advantages. First, it enables the detection of clinically actionable incidental findings. For instance, the *RUNX1* variant establishes a genetic predisposition to familial myeloid neoplasms, delivering a diagnosis that can guides hematological surveillance and cascade testing, a finding that a conventional panel would inherently miss. Second, WES generates a permanent genomic database that allows dynamic computational reanalysis (virtual panels) in response to updates in clinical guidelines or the emergence of new phenotypes, avoiding the “*diagnostic odyssey*” and the need for additional blood draws. Additionally, the accelerated cost reduction between germline panels and WES consolidates the latter as a highly competitive and cost-effective strategy, mitigating the need for sequential testing.

The incidental findings in the *RUNX1, FBXO11* and *FANCA* genes remained within the scope of the research and did not alter the standard cancer treatments guided by the treating oncologists. Also, these genes are not included in the most recent ACMG secondary findings list (ACMG Secondary Findings V3.3, 2025) and adhere to the medical principle “*primum non nocere*: first, do no harm.”

In our pipeline validation cohort, we identified pathogenic variants in the *BRCA1*, *BRCA2*, and *PALB2* genes, which are associated with a high hereditary predisposition to breast and ovarian cancer [30]. These findings are highly actionable. Patients harboring BRCA1/2 or PALB2 mutations derive significant clinical benefit from PARP inhibition. For instance, the *SOLO1* trial demonstrated a ~ 70% reduction in disease progression or death using olaparib in *BRCA1/2*-mutated ovarian cancer, establishing a precedent for germline-directed therapy [31,32]. Additionally, we detected mismatch repair (MMR) defects, specifically *MLH1* and *PMS2* variants consistent with Lynch syndrome. MMR deficiency serves as a robust, tissue-agnostic biomarker for anti-PD-1/PD-L1 immunotherapy. In these tumors, agents like pembrolizumab elicit objective and durable clinical responses regardless of the primary tissue origin, supporting the agnostic indication by biomarker [33,34].

We also identified pathogenic variants in the *STK11* (Peutz-Jeghers syndrome) and *TSC2* (tuberous sclerosis) genes, which are involved in hamartomatous syndromes with the potential for hyperactivation of the mTORC1 pathway. Multisystemic surveillance is recommended for these syndromes, especially for patients with tuberous sclerosis, who may benefit from mTOR-targeted therapies that have demonstrated clinical benefits (such as control of angiomyolipomas and reduction of epileptic seizures with everolimus) [35,36]. In patients with neurocutaneous spectrum disorders, deleterious variants in *NF1* associated with plexiform neurofibromas have demonstrated the sustained benefit of *MEK* inhibition (selumetinib). Similarly, in *NF2*-driven vestibular schwannomas and meningiomas, recent multi-omic studies highlight significant tumor heterogeneity; despite this complexity, maintenance antiangiogenic therapy with bevacizumab provides established clinical benefit in this subgroup [37–39].

In other patients, we identified germline variants in the *TP53*, *EXT1*, and *BAP1* genes. Intensive surveillance with whole-body magnetic resonance imaging is justified in Li-Fraumeni patients with pathogenic variants in *TP53* [40]. In patients with *BAP1* mutations (increases the risk of uveal melanoma, mesothelioma, and renal carcinoma), supported by recent clinical guidelines and genetic counseling, aimed at reducing risk should be provided, such as avoiding occupations with exposure to asbestos, not smoking, and limiting exposure to UV rays [41]. Lastly, regarding *EXT1* variants associated with hereditary multiple exostoses, recent cryo-EM structural resolution of the *EXT1/EXT2* heparan sulfate polymerase complex has significantly advanced the structural basis for the functional interpretation of variants in this gene [42, 43].

In our TNBC discovery cohort (clinicopathological features in S1 Table), we identified three pathogenic variants that support a background of homologous recombination deficiency (HRD) in a subgroup of patients: *BRCA1* (P21), *RAD51D* (P9), and *ATM* (P6). In terms of translational medicine, deleterious *BRCA1/2* variants have strong clinical support for targeted therapies: the OlympiA trial showed a significant benefit of adjuvant olaparib in germinal *BRCA1/2* carriers with high-risk HER2-negative disease. Emerging evidence suggests that alterations in *RAD51* and other HRR genes may define tumors with HRD biology; however, their therapeutic translation must be based on the demonstration of HRD (LOH/TAI/LST, mutational signatures) before extending the benefit of i-PARP beyond patients with pathogenic variants in *BRCA1/2* [32].

The limited size of the cohorts prevents estimating the population-level prevalence of specific genes such as *BRCA1*, *BRCA2*, and *PALB2*. Consequently, the research serves as solid proof of concept and a robust model from a bioinformatics perspective, and makes no claim to be an epidemiological prevalence study. A large-scale cohort is required to provide epidemiological conclusions such as the prevalence of variants and genes involved in hereditary cancer predisposition syndromes in the Latin American population. Due to the nature of the study, the findings did not imply any therapeutic intervention or modification of oncological clinical management based on the patients’ germline variant profiles. Translating these results requires multidisciplinary validation (molecular tumor board), clinical eligibility, and regulatory availability of targeted therapies. Nevertheless, the genomic results are the first step toward advancing precision medicine.

The WES provides a comprehensive landscape of genomic information with high-confidence quality metrics (S2 Table). If a patient tests negative on a standard panel today, future guideline updates or new phenotypic presentations may require additional blood draws, sequencing, and increased costs (“diagnostic odyssey”). With the WES, data can be dynamically reanalyzed bioinformatically (virtual panels) without the need for resequencing. Furthermore, regarding cost-effectiveness, we observe that the sequencing cost difference between a comprehensive germline-targeted panel and the WES is rapidly decreasing, making the WES increasingly competitive and cost-effective, especially by avoiding sequential testing of multiple panels. Additionally, it enables identification of secondary findings in clinically actionable genes recognized by the ACMG. Use of WES supports ongoing precision oncology efforts worldwide, including the identification of tumor-specific variants for neoantigen-based vaccine development, a focus of our work [44]. This research does not aim to describe the epidemiological prevalence of variants or the frequencies of affected genes. Sierra‑Díaz et al. [45] published a profound work about this field on breast cancer in Colombian patients.

In conclusion, we developed a robust bioinformatic pipeline for germline variant identification compliant with GATK Best Practices. Validation against a clinical-grade diagnostic platform confirmed its high accuracy and suitability for variant detection in cancer susceptibility genes from whole-exome sequencing (WES) analysis. By applying this pipeline to our TNBC discovery cohort, we successfully identified causal hereditary cancer variants. Ultimately, this approach allows comprehensive pan-cancer panels end-to-end analysis, impacting patient diagnosis, genetic counseling, and with the potential of guiding precision oncology.

## Supporting information

S1 Table TNBC clinicopathological features. Clinicopathological characteristics summary of the entire triple-negative breast cancer discovery cohort (n = 24), detailing: Age at diagnosis, tumor stage, histology, and treatment.
(XLSX)

S2 Table TNBC quality sequencing metrics. Sequencing metrics summary of the entire triple-negative breast cancer discovery cohort that includes: Patient ID, Average Depth, Hom/Het Ratio, Ti/Tv Ratio, Variant Quality (% reads above Q40), and Total of Variants identified in WES.
(XLSX)

S3 Table Germinal validation. This comparative analysis shows the gold-standard variants identified using clinical bioinformatics platforms and the results validated using the developed bioinformatics pipeline. The genetic variants for each patient include transcript information, genomic coordinates, coding DNA, protein changes, and the ACMG criteria applied for variant classification.
(XLSX)

S4 Table Cancer driver genes comprehensive panel (*pancancer*). Lists of genes with proven clinical and scientific evidence for the development of hereditary cancer (Tier 1), genes recognized as cancer driver genes in the somatic context of tumors (Tier 2), and the union of both lists of genes that constitute the universe described as the comprehensive panel or pancancer panel.
(XLSX)

S5 Table Consolidated TNBC variants. Lists of genetic variants identified in the pancancer panel for all patients with TNBC. The information includes: gene, variation type, chromosome, start and stop position, reference, alteration, dbSNP, transcript, amino acid change, nucleotide, exon, zygosity, region, effect, frequency (gnomAD), *in silico* predictors, and Franklin automated classification.
(XLSX)

S6 Table Pathogenic and likely pathogenic TNBC variants. List of variants classified as pathogenic or likely pathogenic in patients with TNBC and manual curation of these variants strictly applying the *ACMG* criteria. The type and number of SNVs and INDELs variants identified are also included.
(XLSX)

S7 Information Functional implications of the variant in the *ATM* gene. The evidence supports that the variant leads to skipping of exon 14 with disruption of normal gene processing.
(DOCX)
