# Supplementary material for: Exome-based cancer driver gene comprehensive testing can provide a genetic diagnosis for individuals with triple-negative breast cancer
Source: PLoS One. 2026 Aug 24;21(8):e0356762. doi: 10.1371/journal.pone.0356762 (PMC13502651; doi:10.1371/journal.pone.0356762)
Supplement: Supplementary file 1 [file pone.0356762.s007.docx]

***Functional implications of the c.2250G>A variant in the ATM gene***

The synonymous *ATM* variant does not directly alter the amino acid codon 750 of the protein, but causes a substitution of the nucleobase Guanine for Adenine in the last nucleotide of exon 14 of the ATM gene. The NM_000051.4:c.2250G>A variant (p.Lys750=; rs1137887; ClinVar VCV000003044) affects a critical position of the splice donor site (in the literature also referred to as “coding exon 13” or “exon 16” depending on the numbering used). Splicing predictors indicate a strong disruptive or deleterious effect (SpliceAI = 0.94). Consistently, functional studies converge in that *ATM* c.2250G>A (p.Lys750=) alters canonical splicing and causes complete skipping of exon 14, generating the transcript r.2125_2250del and an in-frame deletion of 42 amino acids (p.Glu709_Lys750del) (Sandoval et al. Hum Mol Genet. 1999) [[1]](https://www.zotero.org/google-docs/?VGZ5wG). Sandoval et al. demonstrated by transcript analysis that splice site substitutions produce single-exon skipping; this specific variant (2250G>A) is documented to cause skipping of the corresponding exon and loss of *ATM* function [[1]](https://www.zotero.org/google-docs/?9I0yWO). This effect has been experimentally confirmed by RT-PCR and Sanger studies in a pediatric case and its carrier mother, showing “exon 14 skipping” associated with c.2250G>A and supporting a biallelic loss-of-function mechanism in ataxia-telangiectasia (A-T) (Maroilley et al. Front Genet. 2022) [[2]](https://www.zotero.org/google-docs/?3ipmNg). Consequently, clinical trials summarize the evidence of carrier-derived RNA demonstrating exon 14/16 skipping and the consequent p.(Ile709_Lys750del / Glu709_Lys750del) deletion (ClinVar, VCV000003044), also integrating data on reduced/absent kinase activity and chromosomal instability in cell lines from patients with this alteration (*ClinVar, NCBI Databases*) [[3,4]](https://www.zotero.org/google-docs/?f3itKB). Taken together, functional evidence from multiple sources confirms that c.2250G>A acts as a pathogenic splicing-mediated synonymous variant, with cellular consequences consistent with *ATM* loss of function.

Classic functional studies in lymphoblasts from patients with A-T documented that variants altering this splicing reduce *ATM* expression, decrease kinase activity, and increase chromosomal instability, supporting a damaging effect at the molecular level. Together, the evidence integrates robust in silico prediction, RNA validation, and functional data converging for a pathogenic mechanism of splicing alteration caused by a synonymous variant at the end of the exon. (*Casadei et al. Characterization of splice-altering mutations in inherited predisposition to cancer. PNAS. 2019*) [[5]](https://www.zotero.org/google-docs/?KeK4Zx).

Clinically, c.2250G>A has been repeatedly identified in multiple unrelated individuals with compatible phenotypes (including Ataxia telangiectasia, in homozygous or compound heterozygous states), meeting evidence of case recurrence; additionally, it has been reported in individuals with breast cancer in clinical series and multigene panels. In population-based settings, its frequency is very low (11/250,736 chromosomes in gnomAD v2.1.1) [[6]](https://www.zotero.org/google-docs/?O9Oe5b), consistent with a very rare pathogenic variant. Aggregation of evidence (functional, independent case-based, and splicing prediction) has led multiple laboratories to classify it as pathogenic/likely pathogenic in ClinVar (Variation ID 3044). Taken together, the data support that this synonymous variant acts as a splicing disorder with loss of *ATM* function, relevant to both Ataxia telangiectasia (in biallelicity) and breast cancer predisposition (in heterozygotes).

Importantly, the variant found in the patient's germline meets the American College of Medical Genetics and Genomics criteria: PS3_Strong, PM2, PP3, PP1 (Richards S et al. Genet Med 2015), and can therefore be classified as a likely pathogenic variant (or even pathogenic depending on the weight adjustment on the applied ACMG criteria) [[7]](https://www.zotero.org/google-docs/?NJ9JC9).

Although we did not have peripheral blood RNA seq, we did have the patient's tumor transcriptome. After performing the alignment with the hg38 reference genome using the HISAT2 tool and uploading the RNASeq .bam and .bai files to the IGV visualization tool (Robinson et al., Integrative genomics viewer. Nat Biotechnol. 2011) [[8]](https://www.zotero.org/google-docs/?8f3uaW). Figure A shows the same heterozygous alteration in the last nucleotide of exon 14 in the *ATM* gene (c.2250G>A, p.Lys750=), which demonstrates that this mutation may indeed constitute a mechanism as one of the oncogenic driver genes in the patient's tumor. With the functional evidence described, exon skipping is predicted. Likewise, using the UCSC on Human viewer using the GRCh38/hg38 reference genome (Perez et al., The UCSC Genome Browser database: 2025 update. Nucleic Acids Research 2025) [[9]](https://www.zotero.org/google-docs/?iZvBLr), it can be observed that the 6-nucleotide genomic region in which the alteration is found is a highly conserved region in mammals and with a high intolerance to missense variants, as shown in Figure B.

**Figure A.** Visualization using IGV (hg38) of the tumor transcriptome alignment in the region corresponding to the 3′ end of exon 14 of the *ATM* gene c.2250G>A (p.Lys750=). The mixed orange/green column at the last exonic nucleotide indicates heterozygous expression of the mutant allele with a G>A change at the mRNA level. The position of the mutation is embedded in the exon–intron junction and adjacent junctions, consistent with an altered splicing pattern. Taken together, the figure documents the presence of the mutant allele in tumor RNA and supports its potential impact on ATM splicing.

**Figure B.** UCSC Genome Browser (hg38) capture of the end of exon 14 of the *ATM* gene. Multispecies alignment and sequence logo evidence high conservation of the canonical AG|GT splicing motif at the exon–intron junction, where the exonic terminal nucleotide (G) corresponds to c.2250. Conservation logos (*PhyloP 100 vertebrates)* show elevated peaks at the splice site, consistent with the conservation of K–A–K residues across species. Taken together, the figure illustrates that the last nucleotide of exon 14 resides in a highly conserved context, supporting its functional relevance and the plausibility of impact by changes such as the *ATM* c.2250G>A variant.

The Genomics England PanelApp expert group (Panel 158: Familial Breast Cancer: https://panelapp.genomicsengland.co.uk/panels/158/gene/ATM/) explicitly classifies *ATM* as a 'green list' gene with an autosomal dominant monoallelic inheritance pattern and incomplete penetrance. The panel curators state that loss-of-function (*LoF*) variants in the *ATM* gene confer a 20-30% lifetime risk of breast cancer. This classifies it as a moderate-to-high-risk susceptibility gene [[10]](https://www.zotero.org/google-docs/?Q0f0EE).

In summary, while we have tempered our assertions to acknowledge the *National Comprehensive Cancer Network (NCCN)* designation of the *ATM* gene as a moderate-risk susceptibility gene in breast cancer, the evidence available in the literature, which we also provide with the patient's transcriptome information, strongly supports the pathogenic mechanism of this splicing disorder.

**References**

[1.](https://www.zotero.org/google-docs/?hLsRo4)	[Sandoval N. Characterization of ATM gene mutations in 66 ataxia telangiectasia families. Hum Mol Genet. 1 de enero de 1999;8(1):69-79. doi:10.1093/hmg/8.1.69](https://www.zotero.org/google-docs/?hLsRo4)

[2.](https://www.zotero.org/google-docs/?hLsRo4)	[Maroilley T, Wright NAM, Diao C, MacLaren L, Pfeffer G, Sarna JR, et al. Case Report: Biallelic Loss of Function ATM due to Pathogenic Synonymous and Novel Deep Intronic Variant c.1803-270T > G Identified by Genome Sequencing in a Child With Ataxia–Telangiectasia. Front Genet. 25 de enero de 2022;13:815210. doi:10.3389/fgene.2022.815210](https://www.zotero.org/google-docs/?hLsRo4)

[3.](https://www.zotero.org/google-docs/?hLsRo4)	[Landrum MJ, Lee JM, Riley GR, Jang W, Rubinstein WS, Church DM, et al. ClinVar: public archive of relationships among sequence variation and human phenotype. Nucleic Acids Res. enero de 2014;42(D1):D980-5. doi:10.1093/nar/gkt1113](https://www.zotero.org/google-docs/?hLsRo4)

[4.](https://www.zotero.org/google-docs/?hLsRo4)	[Sayers EW, Beck J, Bolton EE, Brister JR, Chan J, Connor R, et al. Database resources of the National Center for Biotechnology Information in 2025. Nucleic Acids Res. 6 de enero de 2025;53(D1):D20-9. doi:10.1093/nar/gkae979](https://www.zotero.org/google-docs/?hLsRo4)

[5.](https://www.zotero.org/google-docs/?hLsRo4)	[Casadei S, Gulsuner S, Shirts BH, Mandell JB, Kortbawi HM, Norquist BS, et al. Characterization of splice-altering mutations in inherited predisposition to cancer. Proc Natl Acad Sci. 26 de diciembre de 2019;116(52):26798-807. doi:10.1073/pnas.1915608116](https://www.zotero.org/google-docs/?hLsRo4)

[6.](https://www.zotero.org/google-docs/?hLsRo4)	[Karczewski KJ, Francioli LC, Tiao G, Cummings BB, Alföldi J, Wang Q, et al. The mutational constraint spectrum quantified from variation in 141,456 humans. Nature. 28 de mayo de 2020;581(7809):434-43. doi:10.1038/s41586-020-2308-7](https://www.zotero.org/google-docs/?hLsRo4)

[7.](https://www.zotero.org/google-docs/?hLsRo4)	[Richards S, Aziz N, Bale S, Bick D, Das S, Gastier-Foster J, et al. Standards and guidelines for the interpretation of sequence variants: a joint consensus recommendation of the American College of Medical Genetics and Genomics and the Association for Molecular Pathology. Genet Med. mayo de 2015;17(5):405-24. doi:10.1038/gim.2015.30](https://www.zotero.org/google-docs/?hLsRo4)

[8.](https://www.zotero.org/google-docs/?hLsRo4)	[Robinson JT, Thorvaldsdóttir H, Winckler W, Guttman M, Lander ES, Getz G, et al. Integrative genomics viewer. Nat Biotechnol. enero de 2011;29(1):24-6. doi:10.1038/nbt.1754](https://www.zotero.org/google-docs/?hLsRo4)

[9.](https://www.zotero.org/google-docs/?hLsRo4)	[Perez G, Barber GP, Benet-Pages A, Casper J, Clawson H, Diekhans M, et al. The UCSC Genome Browser database: 2025 update. Nucleic Acids Res. 6 de enero de 2025;53(D1):D1243-9. doi:10.1093/nar/gkae974](https://www.zotero.org/google-docs/?hLsRo4)

[10.](https://www.zotero.org/google-docs/?hLsRo4)	[Martin AR, Williams E, Foulger RE, Leigh S, Daugherty LC, Niblock O, et al. PanelApp crowdsources expert knowledge to establish consensus diagnostic gene panels. Nat Genet. noviembre de 2019;51(11):1560-5. doi:10.1038/s41588-019-0528-2](https://www.zotero.org/google-docs/?hLsRo4)
